# Pretreatment with *Saccharomyces boulardii* does not prevent the experimental mucositis in Swiss mice

**DOI:** 10.1186/1477-5751-13-6

**Published:** 2014-04-11

**Authors:** Tatiani Uceli Maioli, Brenda de Melo Silva, Michelle Nobre Dias, Nivea Carolina Paiva, Valbert Nascimento Cardoso, Simone Odilia Fernandes, Cláudia Martins Carneiro, Flaviano dos Santos Martins, Simone de Vasconcelos Generoso

**Affiliations:** 1 Departamento de Nutrição, Escola de Enfermagem, Universidade Federal de Minas Gerais, Belo Horizonte 30130-100, Brazil; 2 Núcleo de Pesquisa em Ciências Biológicas, Instituto de Ciências Exatas e Biológicas, Universidade Federal de Ouro Preto, Ouro Preto 35400-000, Brazil; 3 Departamento de Análises Clínicas e Toxicológicas, Escola de Farmácia, Universidade Federal de Minas Gerais, Belo Horizonte 31270-901, Brazil; 4 Departamento de Microbiologia, Instituto de Ciências Biológicas, Universidade Federal de Minas Gerais, Belo Horizonte 31270-901, Brazil

**Keywords:** Probiotic, Mucositis, 5-fluorouracil, *Saccharomyces boulardii*, Intestinal permeability

## Abstract

**Background:**

The antimetabolite chemotherapy 5-Fluorouracil is one of the most commonly prescribed drugs in clinical cancer treatment. Although this drug is not specific for cancer cells and also acts on healthy cells, it can cause mucositis, a common collateral effect. Dysbiosis has also been described in 5-fluorouracil-induced mucositis and is likely to contribute to the overall development of mucositis. In light of this theory, the use of probiotics could be a helpful strategy to alleviate mucositis. So the aim of this study was evaluate the impact of the probiotic *Saccharomyces boulardii* in a model of mucositis.

**Results:**

After induced of mucositis, mice from the Mucositis groups showed a decrease in food consumption (p < 0.05) and therefore had a greater weight loss (p < 0.05). The treatment with *Saccharomyces boulardii* did not reverse this effect (p > 0.05). Mucositis induced an increase in intestinal permeability and intestinal inflammation (p < 0.05). There were no differences in mucosal lesions, intestinal permeability and sIgA secretion (p > 0.05) in mice pretreated with *S. boulardii.*

**Conclusions:**

*S. boulardii* was not able to prevent the effects of experimental mucositis induced by 5- Fluorouracil.

## Background

The antimetabolite chemotherapy 5-Fluorouracil (5-FU) is one of the most commonly prescribed drugs in clinical cancer treatment [1]. It is used in many types of cancer, including breast, pancreas, gastrointestinal, head and neck [2-4]. Although this drug is not specific for cancer cells and also acts on healthy cells, it can cause mucositis, a common collateral effect of cancer chemotherapy and radiotherapy. This problem can affect the entire gastrointestinal tract, causing odynophagia, vomiting, abdominal pain and diarrhea [5,6]. These symptoms are associated with decreased food consumption, weight loss and diminished nutritional status [7]. Approximately 40% of patients are affected by mucositis when they receive normal doses of chemotherapy, and almost all patients have this problem when they receive higher doses, for example, in cases of treatment for leukemia and bone marrow transplantation [8]. Mucositis has a huge clinical and economic impact because it increases the prevalence of infection and hemorrhage and prolongs the time and cost of hospitalization [6].

Treatment with 5-FU causes significant villus shortening and decreases the villus/crypt ratio in both human and animal models [9,10]. This alteration in the intestinal morphology can disrupt physical barriers and favor the translocation of microorganisms, increasing the possibility of sepsis [11,12].

Dysbiosis has also been described in 5-FU-induced mucositis and is likely to contribute to the overall development of mucositis [13]. In light of this theory, the use of probiotics could be a helpful strategy to alleviate mucositis. Probiotics have been investigated as a therapeutic approach in a range of disorders, including inflammatory bowel disease and other intestinal problems [14,15]. Probiotics are defined as living microorganisms, which when administered in adequate amounts, confer a health benefit to the host [16]. *Saccharomyces boulardii* is probiotic yeast that has been proven to be effective in the treatment of a variety of diarrheal diseases. This yeast has been successfully used for the prevention and treatment of antibiotic-associated diarrhea [17-21].

Previous results of our research showed that *S. boulardii* was able to reduce intestinal permeability and bacterial translocation in a model of intestinal obstruction [14]. To study a novel way to alleviate mucositis, we decided to investigate the effects of probiotic *S. boulardii* in preventing the symptoms caused by 5-FU treatment in an experimental model of mucositis.

## Results

### Food consumption and weight during treatment

**Figure 1:**
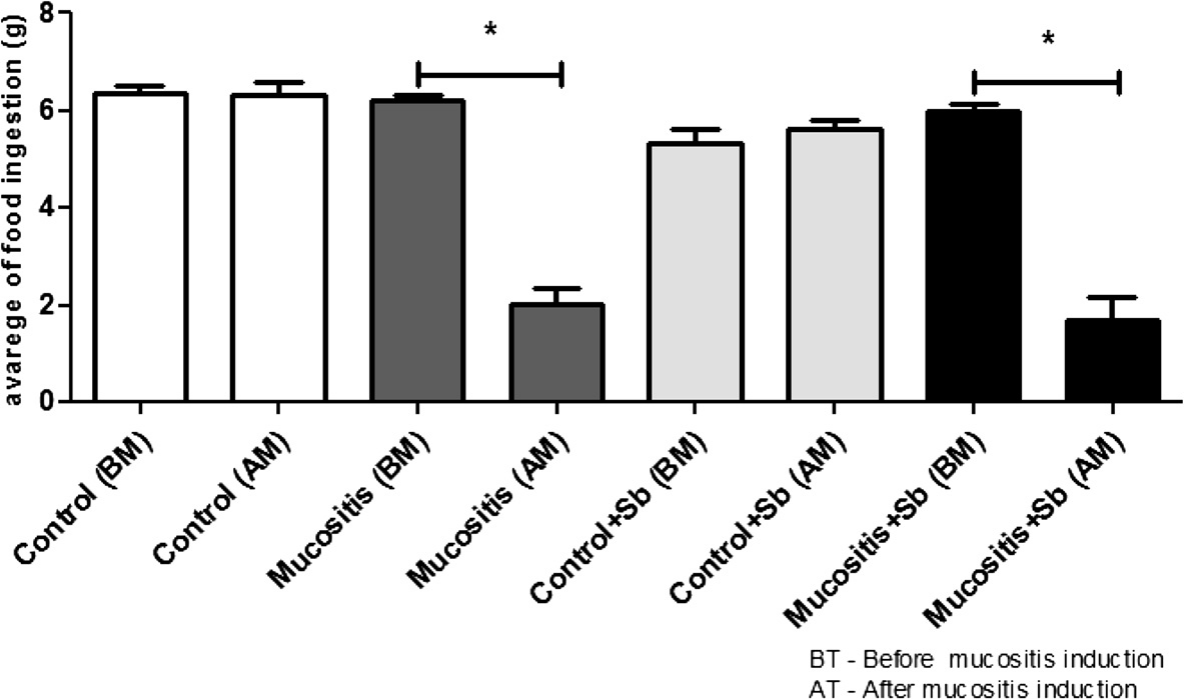
**Variation in food consumption.** Mice received commercial chow *ad libitum* during the entire experiment. The amounts of food consumed were measured every day. (*) Indicates statistically significant differences (p < 0.05), n = 7; data are representative of three different experiments. Sb - *S. boulardii*; BM - before mucositis induction; AM - after mucositis induction.

**Figure 2:**
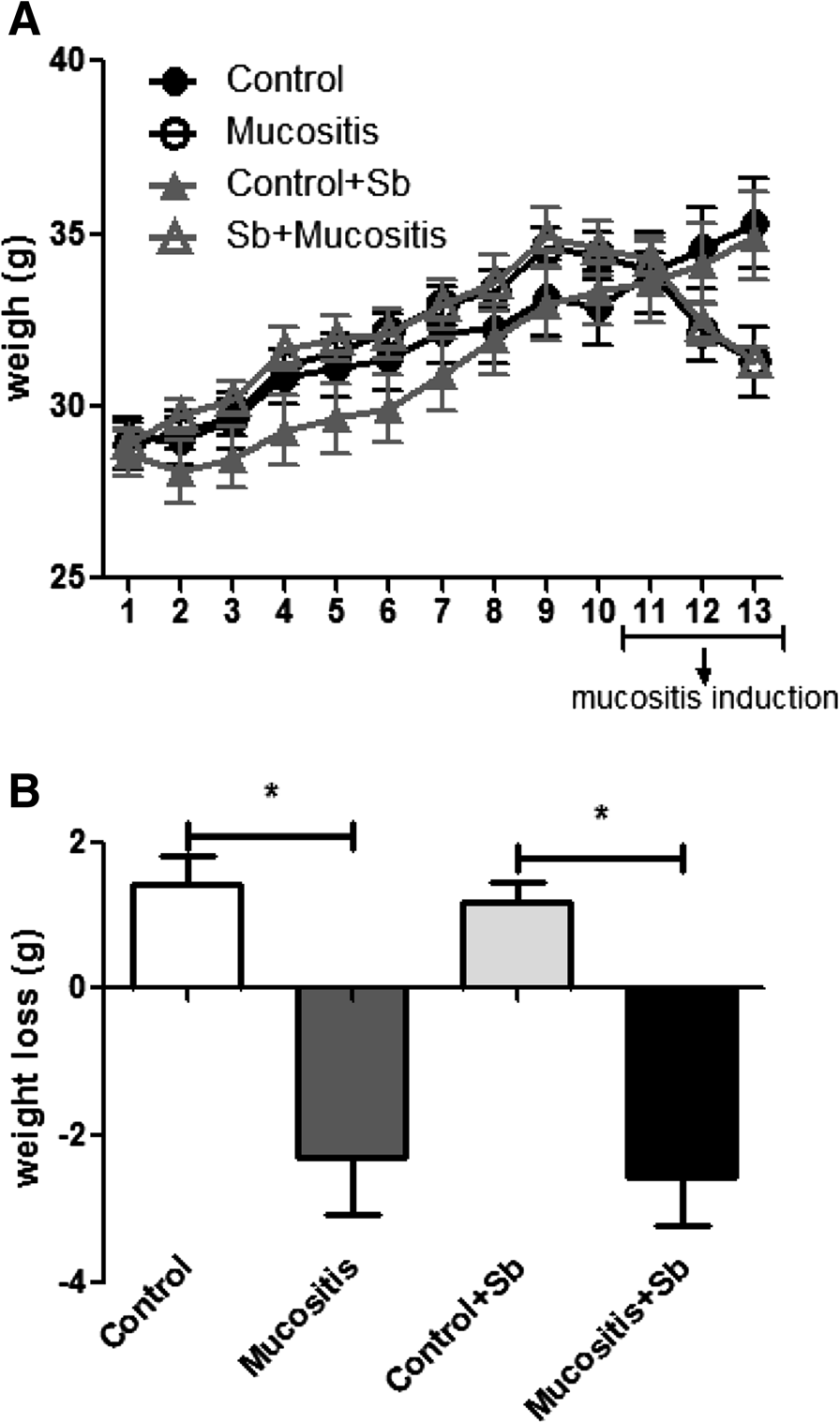
**Weight variation during the experiment.** Mice from the Control + Sb and Mucositis + Sb groups were pre-treated with 10^8^ CFU of *S. boulardii* by gavage for 10 days; other groups received saline by gavage. On day 11 of the experiment, mice from the Mucositis group received an intraperitoneal injection of 300 mg/kg of 5-FU. The weights were monitored daily until day 13 **(A)**, and weight losses represent the difference between the weight on day 11 and day 13 **(B)**. (*) Indicates statistically significant differences (p < 0.05), n = 7; data are representative of three different experiments.

Food consumption and weight gain were measured during the experiments. There was no difference in the amount of food consumed among the groups treated with saline or *S. boulardii* (Sb). After injection of 5-FU, mice from the Mucositis groups showed a decrease in food consumption, and the treatment with Sb did not reverse this effect (Figure 1). As was expected, there were weight losses after mucositis induction (Figure 2A and B). Mice from the Mucositis groups lost approximately 3 g in 3 days (Figure 2B). Pre-treatment with *S. boulardii* did not prevent this effect.

### Intestinal permeability and histological analyses

**Figure 3:**
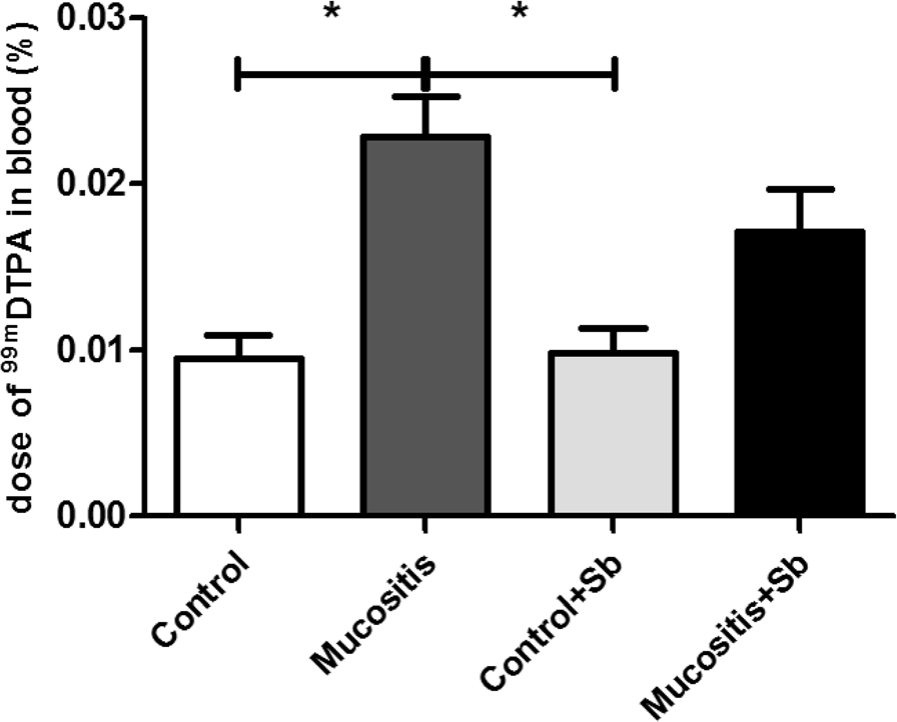
**Effect of pre-treatment with *S. boulardii *on intestinal permeability.** Mice from the Control + Sb and Mucositis + Sb groups were pre-treated with 10^8^ CFU of *S. boulardii* by gavage for 10 days; other groups received saline by gavage. On the 11^th^ day of the experiment, mice from Mucositis group received an intraperitoneal injection of 300 mg/kg of 5-FU. After 72 h, all received ^99m^DNTP by gavage, and four hours later, they were given general anesthesia and had their blood collected. % dose = (counts per minute of blood/counts per minute of administered dose). (*) Indicates statistically significant differences (p < 0.05), n = 7; data are representative of three different experiments.

Intestinal permeability is one of the main problems caused by chemotherapy, including treatment with 5-FU. We investigated whether pre-treatment with Sb could alleviate this problem. Figure 3 shows that 5-FU increased intestinal permeability measured by ^99m^DTPA in the blood. No statistical difference was observed between the Mucositis and Mucositis + Sb groups for this response.

**Figure 4:**
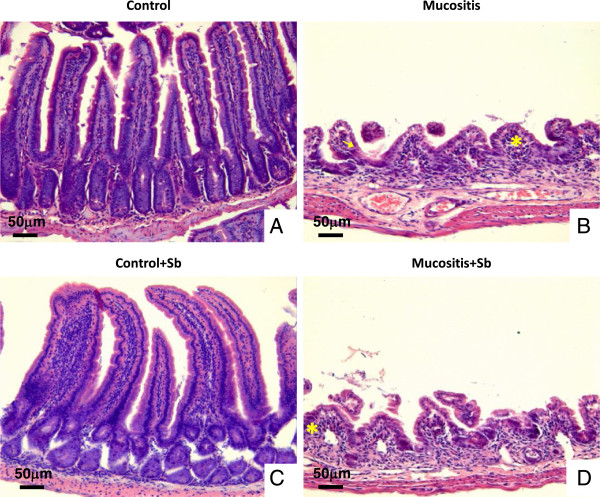
**Histological analyses of slices from the small intestine of mice pre-treated or not with *S. boulardii *and subjected to mucositis induction.** Normal histological aspects in mucosa were observed in mice from the Control and Control + Sb groups **(A and C)**. Increased infiltration of cells in the lamina propria (*) and alterations in the Lieberkün crypt glands (arrow) were observed in mice that developed mucositis **(B and D)**. Bar = 50 μm. The slices were stained with HE. 200x.

Histological analyses were performed to confirm the permeability data. The Control and Control + Sb mice had healthy small intestinal mucosa with no inflammatory infiltration in the mucosal, sub mucosal or muscular layers. The villus/crypt ratio was the same in mice treated with *S. boulardii* and those not treated. Thus, the daily probiotic treatment did not alter gut mucosal morphology (Figure 4A and C). The integrity of the mucosa was lost in mice that received 5-FU (Figure 4B and D). Both Mucositis groups showed lesions in the small intestine, particularly in the jejunum and ileum. They also had marked cell infiltration in the lamina propria, decreased villus size, and Paneth cell hypersecretion, as well as inflammation in the submucosa and muscular layers. These data confirm the effect of 5-FU in mucositis development. There were no significant differences between mice pre-treated or not with *S. boulardii*.

**Figure 5:**
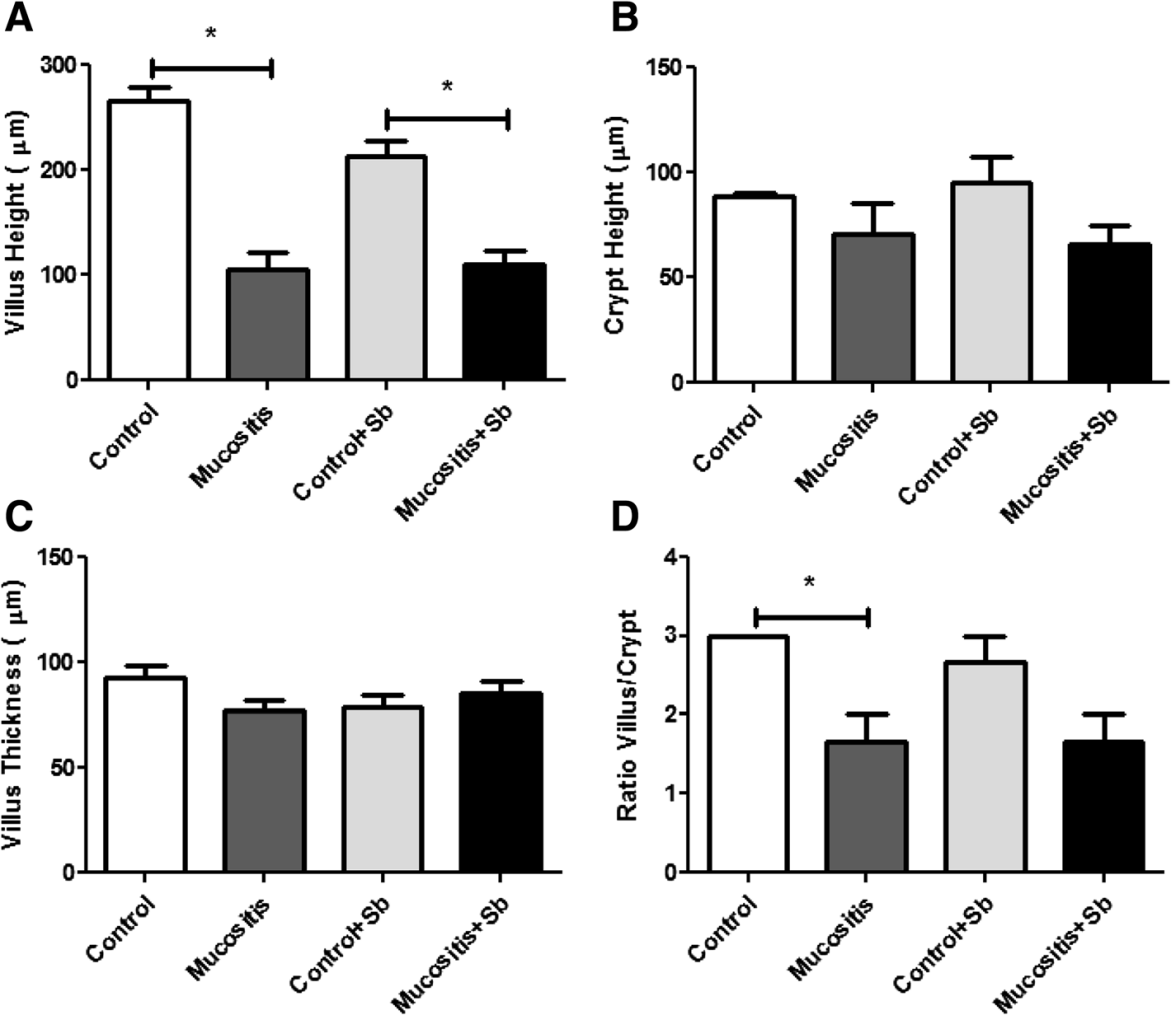
**Morphometrical analyses of small intestine slices from mice pre-treated or not with *S. boulardii *and subjected to mucositis induction.** Mice from the Control + Sb and Mucositis + Sb groups were pre-treated with 10^8^ CFU of *S. boulardii* by gavage for 10 days; other groups received saline by gavage. On the 11^th^ day of the experiment, mice from the Mucositis group received an intraperitoneal injection of 300 mg/kg of 5-FU. **A)** villus mean height (μm), **B)** Lieberkün crypt mean height (μm), **C)** villus mean thickness (μm) and **D)** ratio between villus and crypt mean height. (*) Indicates statistically significant differences (p < 0.05), n = 3.

To confirm the data from the histological slides, morphometric analyses were performed and showed decreases in villus height in both mucositis groups, but there were no statistical differences between the Mucositis and Mucositis + Sb groups (Figure 5A). In none of the groups were differences in crypt height or in villus thickness (Figures 5B and C). There were also no differences in the villus/crypt ratio between control groups and mucositis groups (Figure 5D). These results suggest that 5-FU has more effect on the villus than the crypt.

### IgA secretion

**Figure 6:**
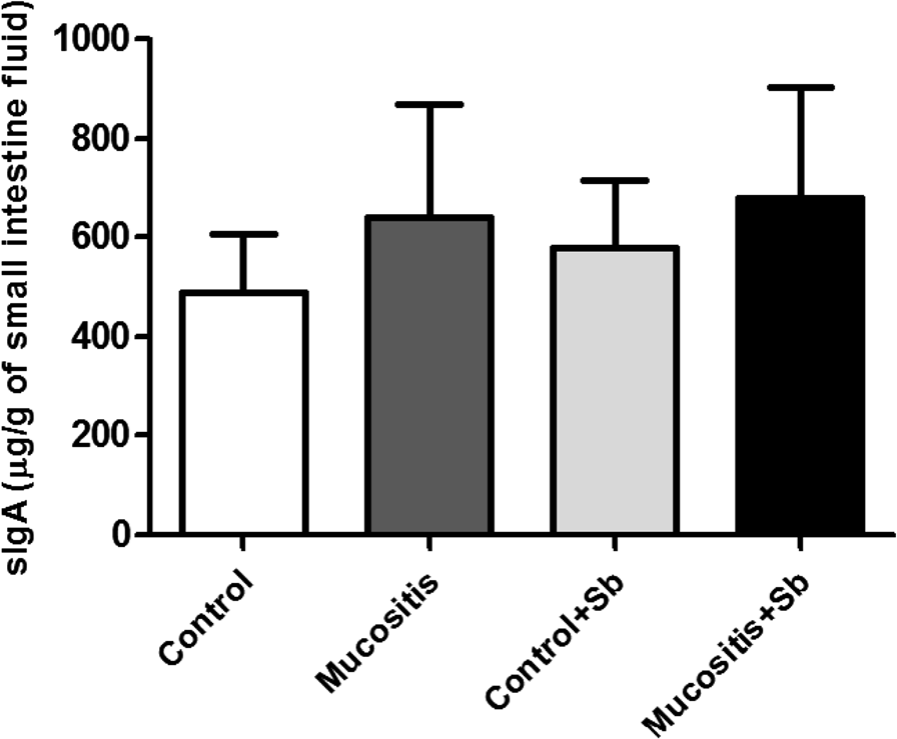
**Secretory IgA in the small intestinesof mice pre-treated or not with *S. boulardii *and subjected to mucositis induction.** Mice from the Control + Sb and Mucositis + Sb groups were pre-treated with 10^8^ CFU of *S. boulardii* by gavage for 10 days; other groups received saline by gavage. On the 11^th^ day of the experiment, mice from the Mucositis group received an intraperitoneal injection of 300 mg/kg of 5-FU. Small intestine fluid was collected and sIgA was measured by ELISA.

IgA secretion in the small intestine was also investigated. We did not observe any statistical differences among the groups (Figure 6).

## Discussion

The current study assessed the effects of treatment with *S. boulardii* on 5-FU induced intestinal damage *in vivo*, an experimental model for mucositis. It has been suggested that this probiotic yeast has beneficial properties, improving the gut immune response and the intestinal barrier [14,17,18,22]. However, our results did not show these beneficial effects in an experimental model of mucositis induced by 5-FU. There were no differences in small intestine lesions and intestinal permeability between mice treated with *S. boulardii* and those not treated.

Only few studies have evaluated the effect of probiotics on mucositis, and in some cases, the results are contradictory [1,23-25]. The differences could be explained by the use of several antineoplastic agents for inducing mucositis, including irinotecan, methotrexate, and 5-FU, each of which has a different mechanism of action.

This study used 5-FU at a dose of 300 mg/kg per animal. This dose was tested in our lab for Swiss mice. The 5-FU is an anti-metabolite, pyrimidine analog. Once absorbed into the cell, 5-FU is bioactivated and inhibits thymidylate synthetase by forming a stable, inactivating ternary complex with the enzyme, thereby inhibiting DNA synthesis. 5-FU also exerts its cytotoxic effects through incorporation into RNA and DNA, triggering apoptosis of cancer cells and cells of healthy tissues with high rates of cell division, including cells in the gut [26,27]. As a consequence, there is increased oxidative stress, activation of transcription factors and increased production of inflammatory cytokines leading to destruction of the intestinal mucosa [28,29].

Our results showed that animals in all 4 groups had similar levels of food intake during the treatment with *S. boulardii* or saline (Figure 1). However, after the induction of mucositis, the animals of the Mucositis and Mucositis + Sb groups had significantly less food intake than the Control groups (Figure 1) and consequently, greater weight loss (Figure 2A). The pretreatment with probiotic had no influence on the food intake and weight loss. Tooley et al [24] found similar results using *Streptococcus thermophilus* as probiotic. In their work, animals that received probiotic treatment and were subjected to mucositis induction showed weight losses similar to those of animals that did not receive the probiotic treatment. These results might be explained by the severe inflammation, lesioning and ulceration that can occur in mucositis induced by 5-FU and can directly affect the food intake [8]. One other possible explanation for this observation is that 5-FU induces a delay in gastric emptying as demonstrated by Soares et al [10].

The integrity of the intestinal barrier is influenced by changes in intestinal permeability, the function of the mucosal immunologic system and cellular homeostasis between the production of new enterocytes and the rate of apoptosis of damaged enterocytes [30]. Mucositis is a complex process that causes intestinal injuries, including alterations in brush-border hydrolase activity, villus heights, crypt depths, and increased apoptosis of crypt cells and intestinal permeability [31].

Intestinal permeability is considered increased when permeation of molecules smaller than 150 Daltons (Da) is observed. In this study, intestinal permeability was evaluated by measuring blood radioactivity after the intake of ^99m^Tc-diethylene-triaminopentaacetate (^99m^Tc-DTPA). This compound is a disodium complex with a molecular weight of 549 Da and a half-life of 6 hours, which satisfies the criteria for a marker that can measure intestinal permeability. In addition, ^99m^Tc-DTPA in the blood is an accurate and simple marker of intestinal permeability and changes in gut function, especially in small animals in which urinary analysis is restricted by volume [32-34].

Results showed that mucositis induced an increase in intestinal permeability (Figure 3). As demonstrated in the same Figure, pretreatment with *S. boulardii* was not able to prevent the increase in intestinal permeability. Consistent with this result, histological analysis (Figure 4) showed that animals subjected to induced mucositis presented histological lesions with increases in the number of cells in the lamina propria, shortened villi, hypersecretion by the Paneth cells and inflammatory infiltrate ranging from mild to intense in the submucosa and muscle layers. In contrast, the control group showed histologically normal mucosa with intact structures and a crypt/villus ratio average of 3:1 as well as healthy submucosa and muscle layers. Pretreatment with probiotic yeast in animals with mucositis did not re-establish the tissue architecture but maintained the histological changes seen in the mucositis group. These data are consistent with Mauger et al. [35] who also found no beneficial effects in a model using probiotics in mucositis induced by 5-FU.

The levels of sIgA were measured, but there were no statistical differences among the groups (Figure 6). This result was not expected because several studies had showed improvement of sIgA production after stimulation with yeast cell wall components [15,36]. However, 5-FU leads to cellular apoptosis, decreasing the number of B lymphocytes and other cells responsible for the induction of IgA secretion in the intestine [31,37,38].

Studies with *S. boulardii* have shown beneficial effects on intestinal diseases due to its anti-inflammatory action [39] and activation of mitogen-activated protein kinases [40,41]. In addition, the recovery of intestinal mucosa from experimental mucositis induced by irinotecan has been demonstrated [42]. There is also evidence of the inhibitory effect of live *Streptococcus thermophilus* TH-4 on crypt fission, suggesting therapeutic utility in the prevention of disorders in mucositis induced by 5-FU [25].

For the current model, a possible explanation for the lack of efficacy of the treatment with *S. boulardii* may be the dosage, duration of treatment or inability of this yeast to interfere in the pathogenesis caused by the drug. Mauger et al. [35] did not find beneficial effects using different species of probiotics (*Lactobacillus fermentum, Lactobacillus rhamnosus* GG*,* and *Bifidobacterium lactis* BB 12) in mucositis induced by 5-FU.

## Conclusion

We conclude that the beneficial effects of probiotics differ depending on the strain. Therefore, each strain should be studied separately to demonstrate its effects. More studies with *S. boulardii* are necessary to elucidate the mechanisms involved in the interaction of this yeast with mammalian cells.

## Methods

### Yeast suspension

Viable *S. boulardii* cells from a lyophilized commercial preparation (Floratil, Merck S.A., Rio de Janeiro, RJ, Brazil) were used after isolation on Sabouraud dextrose agar (Difco, Sparks, MD, USA).

### Animals and experimental design

Swiss male mice from self-breeding colony, weighing between 25 and 35 g, were used in this study. The animals were reared in an open animal cage, and water and standard laboratory chow were given *ad libitum*. This study was approved by the Ethics Committee for Animal Experimentation of the Universidade Federal de Minas Gerais (CETEA/UFMG) and complied with the guidelines recommended by the Institute of Laboratory Animal Resources for the care and use of laboratory animals.

The animals were divided into four groups (each group had 7 animals, data are representative of three different experiments with same n): Control, Control + Sb*,* Mucositis, and Mucositis + Sb. For 10 days, mice from the Control + Sb and Mucositis + Sb groups received 10^9^ CFU/mL of *S. boulardii* suspension in saline by gavage, final volume 0.1 mL. The Control and Mucositis groups received the same volume of saline. Every day, the food consumption was obtained by the difference between the amount of offered chow and the residual chow. Individual amount of food ingested were calculated from media of each cage. The weight of the mice were measured every day with a semi analytical balance.

### Mucositis induction with 5-FU

Ten days after treatment with *S. boulardii,* the animals of the Mucositis and Mucositis + Sb groups received an intraperitoneal injection of 300 mg/kg of 5-FU to induce mucositis. The Control and Control + Sb groups received an intraperitoneal injection of the same volume of sterile saline. For three days, the animals continued receiving saline or probiotic by gavage.

### Determination of intestinal permeability

Intestinal permeability was determined by measuring radioactivity diffusion in the blood after oral administration of diethylenetriaminepenta acetic acid (DTPA) labeled with ^99m^Tc. After 10 days of treatment, all mice received 0.1 mL of DTPA solution labeled with 18.5 MBq of ^99m^Tc by gavage. Four hours after gavage, all animals were anesthetized, and their blood was collected and placed in appropriate tubes for radioactivity determinations [14]. Data were expressed as % dose, using the following equation: % Dose = [(CPM of blood/CPM of administered dose)] × 100.

### Histopathological analyses

Animals were euthanized, all the small intestines were collected, and rolls were made. The rolls were fixed with 4% buffered formaldehyde. The material was then embedded in paraffin, and a 4 μm section of each sample was placed on a glass slide and stained with hematoxylin and eosin (HE). The histological characteristics evaluated included the general description of the section. Morphometric analyses were used to check for inflammatory infiltration in the lamina propria, hypersecretionin the Paneth cells, hyperplasia of the goblet cells, submucosal and muscle layers, and general mucosal damage. In addition, the villi and crypts were measured. The villus height/width ratio and the villus height⁄ crypt height ratio from intestinal epithelium were also obtained.

### Intestinal secretory immunoglobulin A (sIgA)

After the mice had been euthanized, the small intestine of each mouse was removed, and the contents were withdrawn, weighed, and suspended in PBS supplemented with an anti-protease cocktail using 500 mg of intestinal contents per 2.0 mL. After centrifugation at 2,000 g for 30 min at 4°C, the supernatant was collected and kept frozen at -80°C until use. Immunoglobulin levels in the intestinal fluids were evaluated by ELISA using goat anti-mouse IgA (Sigma Chemicals Co., St. Louis, MO, USA) and horseradish peroxidase-conjugated goat anti-mouse IgA (Sigma Chemical Co.). Color was developed with o-phenylenediamine (OPD, Sigma Chemical Co.), and absorbance at 492 nm was determined with an ELISA plate reader. The concentrations of the immunoglobulin were determined using a purified mouse IgA standard (Southern Biotechnology Associates, Inc.).

### Statistical analysis

All experiments were performed at least twice. Results from groups were compared using ANOVA with the Dunn test. The differences were considered statistically significant for p < 0.05. All analyses were performed using the program Graph Pad Prism.

## Abbreviations

Da: Daltons; DTPA: Diethylenetriaminepenta acetic acid; 5-FU: 5-Fluorouracil; HE: Hematoxylin and eosin; sIgA: Intestinal secretory immunoglobulin A; Sb: *Saccharomyces boulardii*; 99mTc-DTPA: ^99m^Tc-diethylene-triaminopentaacetate.

## Competing interests

All the authors declare that they have non-financial competing interests.

## Authors’ contributions

TU, BM, MN, NC, VN, SO, CM, FS and SV designed the study and accumulated the data. TU, BM, MN and SV analyzed and interpreted the data and drafted the manuscript. All authors read and approved the final manuscript.

## References

[B1] Prisciandaro LD Geier MS Chua AE Butler RN Cummins AG Sander GR Howarth GS Probiotic factors partially prevent changes to caspases 3 and 7 activation and transepithelial electrical resistance in a model of 5-fluorouracil-induced epithelial cell damage Support Care Cancer 2012 20 3205 3210 10.1007/s00520-012-1446-3 22526145

[B2] Harris DJ Cancer treatment-induced mucositis pain: strategies for assessment and management Ther Clin Risk Manag 2006 2 251 258 10.2147/tcrm.2006.2.3.251 18360600 PMC1936261

[B3] Harris SM Mistry P Freathy C Brown JL Charlton PA Antitumour activity of XR5944 in vitro and in vivo in combination with 5-fluorouracil and irinotecan in colon cancer cell lines Br J Cancer 2005 92 722 728 10.1038/sj.bjc.6602403 15700035 PMC2361868

[B4] Lopes NN Plapler H Lalla RV Chavantes MC Yoshimura EM da Silva MA Alves MT Effects of low-level laser therapy on collagen expression and neutrophil infiltrate in 5-fluorouracil-induced oral mucositis in hamsters Lasers Surg Med 2010 42 546 552 10.1002/lsm.20920 20662031

[B5] Gibson RJ Keefe DM Cancer chemotherapy-induced diarrhoea and constipation: mechanisms of damage and prevention strategies Support Care Cancer 2006 14 890 900 10.1007/s00520-006-0040-y 16604351

[B6] Keefe DM Gibson RJ The combination of oral and small intestinal mucositis, pediatrics and biomarkers: a particularly tricky problem! Cancer Biol Ther 2006 5 1282 1284 10.4161/cbt.5.10.3508 17172807

[B7] Barasch A Peterson DE Risk factors for ulcerative oral mucositis in cancer patients: unanswered questions Oral Oncol 2003 39 91 100 10.1016/S1368-8375(02)00033-7 12509961

[B8] Scully C Epstein J Sonis S Oral mucositis: a challenging complication of radiotherapy, chemotherapy, and radiochemotherapy. Part 2: diagnosis and management of mucositis Head Neck 2004 26 77 84 10.1002/hed.10326 14724910

[B9] Fata F Ron IG Kemeny N O’Reilly E Klimstra D Kelsen DP 5-fluorouracil-induced small bowel toxicity in patients with colorectal carcinoma Cancer 1999 86 1129 1134 10.1002/(SICI)1097-0142(19991001)86:7<1129::AID-CNCR5>3.0.CO;2-4 10506695

[B10] Soares PM Mota JM Gomes AS Oliveira RB Assreuy AM Brito GA Santos AA Ribeiro RA Souza MH Gastrointestinal dysmotility in 5-fluorouracil-induced intestinal mucositis outlasts inflammatory process resolution Cancer Chemother Pharmacol 2008 63 91 98 10.1007/s00280-008-0715-9 18324404

[B11] Gibson RJ Gut microbiome and intestinal mucositis: a new challenge for researchers Cancer Biol Ther 2009 8 512 513 10.4161/cbt.8.6.7852 19411862

[B12] Thom KA Kleinberg M Roghmann MC Infection Prevention in the Cancer Center Clin Infect Dis 2013 4 579 585 23652528 10.1093/cid/cit290 PMC3726067

[B13] Stringer AM Gibson RJ Logan RM Bowen JM Yeoh AS Hamilton J Keefe DM Gastrointestinal microflora and mucins may play a critical role in the development of 5-Fluorouracil-induced gastrointestinal mucositis Exp Biol Med (Maywood) 2009 234 430 441 10.3181/0810-RM-301 19176868

[B14] Generoso SV Viana M Santos R Martins FS Machado JA Arantes RM Nicoli JR Correia MI Cardoso VN Saccharomyces cerevisiae strain UFMG 905 protects against bacterial translocation, preserves gut barrier integrity and stimulates the immune system in a murine intestinal obstruction model Arch Microbiol 2010 192 477 484 10.1007/s00203-010-0574-8 20437166

[B15] Generoso SV Viana ML Santos RG Arantes RM Martins FS Nicoli JR Machado JA Correia MI Cardoso VN Protection against increased intestinal permeability and bacterial translocation induced by intestinal obstruction in mice treated with viable and heat-killed Saccharomyces boulardii Eur J Nutr 2011 50 261 269 10.1007/s00394-010-0134-7 20936479

[B16] FAO FaAOotUNaW, World Health Organization Guidelines For The Evaluation Of Probiotics In Food, Food And Agriculture Organization Of The United Nations & World Health Organization Working Group Report Book Guidelines For The Evaluation Of Probiotics In Food, Food And Agriculture Organization Of The United Nations & World Health Organization Working Group Report (Editor Ed.^Eds.) 2002 City: FAO/WHO

[B17] McFarland LV Meta-analysis of probiotics for the prevention of antibiotic associated diarrhea and the treatment of Clostridium difficile disease Am J Gastroenterol 2006 101 812 822 10.1111/j.1572-0241.2006.00465.x 16635227

[B18] Liong MT Safety of probiotics: translocation and infection Nutr Rev 2008 66 192 202 10.1111/j.1753-4887.2008.00024.x 18366533

[B19] Elmer GW McFarland LV Biotherapeutic agents in the treatment of infectious diarrhea Gastroenterol Clin North Am 2001 30 837 854 10.1016/S0889-8553(05)70213-2 11586560

[B20] D’Souza AL Rajkumar C Cooke J Bulpitt CJ Probiotics in prevention of antibiotic associated diarrhoea: meta-analysis BMJ 2002 324 1361 10.1136/bmj.324.7350.1361 12052801 PMC115209

[B21] Czerucka D Rampal P Experimental effects of Saccharomyces boulardii on diarrheal pathogens Microbes Infect 2002 4 733 739 10.1016/S1286-4579(02)01592-7 12067833

[B22] Buts JP De Keyser N Effects of Saccharomyces boulardii on intestinal mucosa Dig Dis Sci 2006 51 1485 1492 10.1007/s10620-005-9016-x 16838119

[B23] Prisciandaro LD Geier MS Butler RN Cummins AG Howarth GS Probiotic factors partially improve parameters of 5-fluorouracil-induced intestinal mucositis in rats Cancer Biol Ther 2011 11 671 677 10.4161/cbt.11.7.14896 21307648

[B24] Tooley KL Howarth GS Lymn KA Lawrence A Butler RN Oral ingestion of Streptococcus thermophilus does not affect mucositis severity or tumor progression in the tumor-bearing rat Cancer Biol Ther 2011 12 131 138 10.4161/cbt.12.2.15720 21508671

[B25] Whitford EJ Cummins AG Butler RN Prisciandaro LD Fauser JK Yazbeck R Lawrence A Cheah KY Wright TH Lymn KA Howarth GS Effects of Streptococcus thermophilus TH-4 on intestinal mucositis induced by the chemotherapeutic agent, 5-Fluorouracil (5-FU) Cancer Biol Ther 2009 8 505 511 10.4161/cbt.8.6.7594 19305160

[B26] Jolivet J Dayan A Beauchemin M Chahla D Mamo A Bertrand R Biochemical and Molecular Studies of Human Methenyltetrahydrofolate Synthetase Oncologist 1996 1 248 254 10387998

[B27] Longley DB Harkin DP Johnston PG 5-fluorouracil: mechanisms of action and clinical strategies Nat Rev Cancer 2003 3 330 338 10.1038/nrc1074 12724731

[B28] Longley DB Latif T Boyer J Allen WL Maxwell PJ Johnston PG The interaction of thymidylate synthase expression with p53-regulated signaling pathways in tumor cells Semin Oncol 2003 30 3 9 12802789 10.1016/s0093-7754(03)00119-2

[B29] Sonis ST Fey EG Oral complications of cancer therapy Oncology (Williston Park) 2002 16 680 686 discussion 686, 691-682, 695 12108892

[B30] Howarth GS Wang H Role of endogenous microbiota, probiotics and their biological products in human health Nutrients 2013 5 58 81 10.3390/nu5010058 23306189 PMC3571638

[B31] Li X Slayton WB Molecular mechanisms of platelet and stem cell rebound after 5-fluorouracil treatment Exp Hematol 2013 7 635 645,e3 23507524 10.1016/j.exphem.2013.03.003

[B32] dos Santos R Viana ML Generoso SV Arantes RE Davisson Correia MI Cardoso VN Glutamine supplementation decreases intestinal permeability and preserves gut mucosa integrity in an experimental mouse model JPEN J Parenter Enteral Nutr 2010 34 408 413 10.1177/0148607110362530 20631386

[B33] Katouzian F Sblattero D Not T Tommasini A Giusto E Meiacco D Stebel M Marzari R Fasano A Ventura A Dual sugar gut-permeability testing on blood drop in animal models Clin Chim Acta 2005 352 191 197 10.1016/j.cccn.2004.09.023 15653114

[B34] Sun Z Wang X Andersson R Role of intestinal permeability in monitoring mucosal barrier function. History, methodology, and significance of pathophysiology Dig Surg 1998 15 386 397 10.1159/000018651 9845620

[B35] Mauger CA Butler RN Geier MS Tooley KL Howarth GS Probiotic effects on 5-fluorouracil-induced mucositis assessed by the sucrose breath test in rats Dig Dis Sci 2007 52 612 619 10.1007/s10620-006-9464-y 17237997

[B36] Qamar A Aboudola S Warny M Michetti P Pothoulakis C LaMont JT Kelly CP Saccharomyces boulardii stimulates intestinal immunoglobulin A immune response to Clostridium difficile toxin A in mice Infect Immun 2001 69 2762 2765 10.1128/IAI.69.4.2762-2765.2001 11254650 PMC98222

[B37] Tsuboi I Hirabayashi Y Harada T Koshinaga M Kawamata T Kanno J Inoue T Aizawa S Role of hematopoietic microenvironment in prolonged impairment of B cell regeneration in age-related stromal-cell-impaired SAMP1 mouse: effects of a single dose of 5-fluorouracil J Appl Toxicol 2008 28 797 805 10.1002/jat.1341 18344199

[B38] Vetvicka V Kincade PW Witte PL Effects of 5-fluorouracil on B lymphocyte lineage cells J Immunol 1986 137 2405 2410 3093575

[B39] Thomas S Metzke D Schmitz J Dorffel Y Baumgart DC Anti-inflammatory effects of Saccharomyces boulardii mediated by myeloid dendritic cells from patients with Crohn’s disease and ulcerative colitis Am J Physiol Gastrointest Liver Physiol 2011 301 G1083 1092 10.1152/ajpgi.00217.2011 21903765

[B40] Martins FS Vieira AT Elian SD Arantes RM Tiago FC Sousa LP Araujo HR Pimenta PF Bonjardim CA Nicoli JR Teixeira MM Inhibition of tissue inflammation and bacterial translocation as one of the protective mechanisms of Saccharomyces boulardii against Salmonella infection in mice Microbes Infect 2013 15 270 279 10.1016/j.micinf.2012.12.007 23376166

[B41] Thomas S Przesdzing I Metzke D Schmitz J Radbruch A Baumgart DC Saccharomyces boulardii inhibits lipopolysaccharide-induced activation of human dendritic cells and T cell proliferation Clin Exp Immunol 2009 156 78 87 10.1111/j.1365-2249.2009.03878.x 19161443 PMC2673744

[B42] Sezer A Usta U Cicin I The effect of Saccharomyces boulardii on reducing irinotecan-induced intestinal mucositis and diarrhea Med Oncol 2009 26 350 357 10.1007/s12032-008-9128-1 19067257

